# Comparison of DNA and RNA, and Cultivation Approaches for the Recovery of Terrestrial and Aquatic Fungi from Environmental Samples

**DOI:** 10.1007/s00284-012-0256-7

**Published:** 2012-10-27

**Authors:** Subramanya Rao, Kevin D. Hyde, Stephen B. Pointing

**Affiliations:** 1 School of Biological Sciences, The University of Hong Kong, Pokfulam Road, Hong Kong SAR, China; 2 School of Science, Mae Fah Luang University, Chiang Rai, 57100 Thailand

## Abstract

Estimates of fungal biodiversity from environmental samples are all subject to bias. Major issues are that the commonly adopted cultivation-based approaches are suitable for taxa which grow readily under laboratory conditions, while the DNA-based approaches provide more reliable estimates, but do not indicate whether taxa are metabolically active. In this study, we have evaluated these approaches to estimate the fungal diversity in soil and freshwater samples from a subtropical forest, and compared these to RNA-based culture-independent approach intended to indicate the metabolically active fungal assemblage. In both soil and freshwater samples, the dominant taxon recovered by all three approaches was the same (*Anguillospora furtiva*). This taxon was cultivable from all samples and comprised 85–86 % DNA libraries and 90–91 % RNA libraries. The remaining taxa were phylogenetically diverse and spanned the Ascomycota, Basidiomycota, and Fungi *incertae sedis*. Their recovery was not consistent among the three approaches used and suggests that less abundant members of the assemblage may be subjected to greater bias when diversity estimates employ a single approach.

**Electronic supplementary material:**

The online version of this article (doi:10.1007/s00284-012-0256-7) contains supplementary material, which is available to authorized users.

## Introduction

Hong Kong, which is an island located on the southern coast of China, historically supported a dense monsoon forest [10, 14]. The forest has been disappearing since the 19th century due to human exploitation for urbanization and forest fires [14, 27]. Recently, to promote forest conservation and sustainable management, the Hong Kong government has taken an initiative for the re-plantation and protection of forests near Tai Po Kau. The main tree used for re-plantation was the Chinese red pine *Pinus* *massoniana* [2]. Furthermore, many native species of plants are also present in this area.

Soil fungal assemblages are essential components of nutrient cycles and display an important functional role in the forest ecosystem [15]. Though the ecological features of fungi, both in aquatic and terrestrial habitats are well documented [17], the fungal community structure and their dynamics is known relatively less [7]. Previous studies of Ingoldian fungi in Tai Po Kau forest by traditional cultivation methods revealed diverse taxa [9]. However, molecular methods have now become a standard approach in microbiology and have been widely exploited [3–8, 16, 19–23]. Environmental DNA-based analysis in the past has revealed fungal diversity in forest ecosystems; however, it must be noted that the DNA can persist for species which are metabolically inactive and functionally less important [21, 22]. This can potentially be addressed by targeting RNA molecules which are transcribed in metabolically active cells [4, 6, 7, 20, 28].

Recent studies have employed high throughput sequencing to understand forest soil fungal diversity and revealed the richness and identity of the fungal species in the forest ecosystem [8, 19]. However, a key issue in soil mycological studies is to detect what fungal fractions are metabolically active, rather than detecting what fungal signatures are recoverable [23]. By studying the active fungal assemblages, it is possible to understand the forest soil fungal ecology in a better manner. Thus, in this study, we have employed direct extraction of RNA from environmental samples which was subjected to cDNA synthesis via reverse transcription (RT) polymerase chain reaction (PCR) followed by community profiling by means of terminal restriction fragment length polymorphism (T-RFLP) to display the community structure of active fungi in the forest ecosystem. The precursor internal transcribed spacer (ITS) region of the ribosomal RNA gene can be detected in the RNA pools from the soil fungi [4]. In addition, it is also proposed that ITS rRNA gene region reflects more active part of the fungal community and hence has been widely used to detect the soil fungal activity [4, 7, 22, 23]. Here, we have employed and analyzed three different approaches, viz., cultivation, DNA- and RNA-based strategies, for the identification of Tai Po Kau forest fungal species by targeting ITS rRNA gene region.

## Materials and Methods

### Soil Sampling

Samples were collected in sterile tubes from aquatic stream and terrestrial habitat in the Tai Po Kau forest, Hong Kong. Twenty samples were collected comprising equal number of samples from freshwater sediment and terrestrial soils. The collected samples were stored in RNA*later* (Ambion, USA) nucleic acid preservative solution at ambient temperature until processed.

### Recovery of Environmental RNA, DNA, and PCR Amplification

Total RNA was extracted from 50 mg of soil using TRI reagent (Molecular Research Centre, Inc.) and quantified by spectrophotometry (Smartspec-Plus, Bio-Rad, California). The cDNA was synthesised using 1–2 μl (50–100 ng) of RNA, 0.5 μg of oligo (dT)_15_ and ImProm-II™ Reverse transcriptase (Promega, USA). Total DNA was extracted from 50 mg of soil using PowerSoil™ DNA isolation kit following the manufacturer’s protocol (MO BIO Laboratories Inc., Carlsbad, CA, USA). DNA amplification was performed by PCR. The ITS5 and ITS4 [29] primer pair were used to amplify ITS rRNA gene region. The temperature profile included an initial denaturation step at 95 °C for 3 min followed by 34 cycles comprising of denaturation at 95 °C for 1 min, a primer annealing step at 52 °C for 50 s, and an extension step at 72 °C for 1 min. A final extension at 72 °C for 10 min was done at the end of the PCR amplification and the reaction was held at 4 °C until further processing. The presence of PCR products was confirmed by electrophoresis in 1 % agarose gels. Purification of PCR product was carried out using GFX™ PCR DNA and gel band purification kit (GE healthcare, United Kingdom).

### T-RFLP analysis

An initial assessment of fungal diversity in ten soil samples was made by T-RFLP signatures of the ITS region. T-RFLP analysis quantifies the sequence variability in ITS rRNA and produces a unique DNA fingerprint for each fungal community respectively. T-RFLP PCR was carried out using fluorescently labeled primer ITS5 and non-labeled reverse primer ITS4. Purification of PCR products was done as already mentioned above. Restriction digests (*Hin*fI, *Cfo*I) of 6-carboxyfluorescein (FAM)-labeled PCR products were subjected to fragment analysis using 3730 Genetic Analyzer (Applied Biosystems). Statistical analysis was carried out as outlined in [1]; Nonmetric multidimensional scaling ordination (NMDS) were plotted by means of Primer v6.1.6 [11].

### Clone Library Construction and Sequencing

Clone libraries (*n* = 100) were constructed for RNA (cDNA) and DNA using the TOPO TA Cloning^®^ kit (Invitrogen). Automated DNA sequencing was performed using the 3730 Genetic Analyzer (Applied Biosystems). Phylotypes were delineated on the basis of 97 % sequence similarity by the freeware DOTUR (www.mothur.org/software/dotur.html) [24]. Sampling effort was assessed by the calculation of rarefaction curves and estimation of OTU richness from clone libraries were made using Chao1 with EstimateS (http://viceroy.eeb.uconn.edu/estimates/) [12]. Approximate phylogenetic affiliations were determined by BLAST searches on NCBI GenBank database (http://www.ncbi.nlm.nic.gov/).

### Sequence Alignment and Phylogenetic Analyses

Sequences obtained from the respective RNA and DNA samples were used to create multiple sequence alignments with selected GeneBank sequences as references by ClustalX v.1.81 [26]. Maximum likelihood analysis was performed by PAUP* 4.0b8 [25]. Bootstrap values (1,000 replications) are shown for branch nodes supported by more than 70 %. All sequences have been deposited in the NCBI GenBank database under accession numbers JF831451–JF831506, JN409347, and JN409348.

### Cultivation of Fungi from Freshwater Sediment and Terrestrial Soil

Fungi were isolated by diluting 1 g of soil sample in 100 ml of sterile water. Aliquots of 0.5 ml were spread plated onto 2 % Difco MEA (malt extract agar), PDA (potato dextrose agar), and SDA (Saboraud-Dextrose-Agar). Direct soil plating was also carried out, where 0.5 g of soil was directly introduced to agar surfaces. The plates were sealed and incubated at room temperature for 7 days. For each treatment, one uninoculated control plate was also incubated to allow detection of putative contaminants.

### Phylogenetic Analysis of Cultivated Strains

The genomic DNA was isolated from the cultivated fungi by the cetyl trimethylammonium bromide (CTAB) method and quantified by spectrophotometry [18]. The ITS rRNA gene was amplified and sequenced as described above. The phylogenetic analysis based on ITS rRNA gene sequences was performed as described above.

## Results and Discussion

### Fungal Community Profiling

**Fig. 1:**
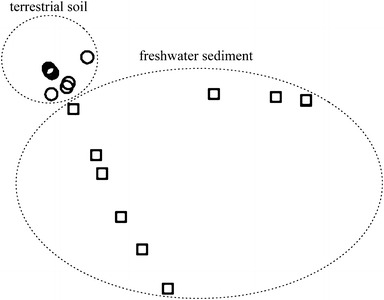
NMDS plots produced by T-RFLP analysis of ITS sequences from environmental RNA (cDNA) from the freshwater sediment (*squares*) and soil (*circles*)

T-RFLP analysis showed a marked variation in fungal communities among fresh water sediment and terrestrial soil samples (Fig. 1). Fungal communities primarily clustered according to their habitat (aquatic or terrestrial). The difference among fungal community was distinct across aquatic sediment and terrestrial soils and was statistically significant (ANOSIM, Global *R* = 0.039, *P* < 0.001).

### Aquatic Fungi

In fresh-water sediment, cultivation methods revealed 11 fungal taxa, comprising *Anguillospora furtiva*, *Trichoderma atroviridae, T*. *velutinum, T*. *brevicompactum, Trichoderma* sp., *Hypoxylon* sp., *Hypocrea lixii, Mortierella elongata, Mortierella* sp. unidentified Mucoromycotina species and unidentified Ascomycota species (Fungal sp. ARIZ) (Table 1). Our DNA-based analysis of the same fresh-water sediment sample, using a clone library (*n* = 100) yielded six phylotypes in the DNA library (Supplementary Fig. 1). A BLAST search of the NCBI GenBank database indicated that over 86 % of the phylotypes were affiliated with *A*. *furtiva*. The remaining phylotypes were affiliated with *T*. *atroviridae* (4 %), *Mortierella* sp. (4 %), *M*. *elongata* (1 %), *Fungal* sp. ARIZ (1 %), *T*. *spirale* (1 %), *Aquaticola hongkongensis* (1 %), and *Cryptococcus folicola* (1 %). The second library was constructed from cDNA, synthesized from environmental RNA (*n* = 100) and revealed three phylotypes in the cDNA library (Supplementary Fig. 1). The BLAST search indicated that almost over 91 % of phylotypes were showing affiliation with *A*. *furtiva*. The remaining phylotypes were affiliated with *T*. *spirale* (3 %), *T*. *atroviridae* (2 %), *T*. *velutinum* (2 %), Fungal sp. ARIZ (1 %) and *Trichoderma* sp. (1 %).

**Table1:** Summary of the relative abundance of fungal phylotypes in environmental RNA and DNA clone libraries of fungal ITS loci, from the freshwater sediment of Tai Po Kau forest

S.No.	Best BLAST match	Culture		DNA (%)	RNA (%)	Sample label culture/DNA/RNA	Accession no.
Ascomycota							
1	*Anguillospora furtiva* (AY148107)	√		86	91	IPDA9/IDNA6/IcDNA7	JF831453
2	Fungal sp. ARIZ (FJ612844)	√		1	1	-/IDNA113/IcDNA98	JF831489
3	*Trichoderma atroviride* (HQ259980)	√		4	2	-/IDNA41/IcDNA18	JF831475
4	*Trichoderma velutinum* (HM176574)	√		0	2	IMEA6/-/IcDNA57	JF831476
5	*Trichoderma brevicompactum* (EU280088)	√		0	0	–	–
6	*Trichoderma spirale* (EU280068)	×		1	3	-/IDNA101/IcDNA10	JF831490
7	*Trichoderma* sp. (EU082794)	√		0	1	IMEA5/-	JF831479
8	*Hypoxylon* sp. (GQ334435)	√		0	0	IMEA3/-	JF831496
9	*Aquaticola hongkongensis* (AF177156)	×		1	0	-/IDNA24/-	JF831462
10	*Hypocrea lixii* (AB570245)	√		1	0	IPDA2/IDNA93/-	JF831464
Basidiomycota							
11	*Cryptococcus folicola* (AY557600)	×		1	0	-/IDNA42/-	JF831497
Fungi *incertae sedis*							
12	*Mortierella elongata* (AB542111)	√		1	0	/-IDNA19/-	JF831500
13	*Mortierella* sp. (EU877758)	√		4	0	-/IDNA39/-	JF831498
14	*Mucoromycote* sp. (EU076939)	√		0	0	IMEA4/-/-	JF831506

The polyphasic nature of our investigation on fresh-water sediments indicated that the most common fungus was *A*. *furtiva*, which was also the dominant fungus in aquatic sediments, and was recovered with all three approaches. However, Fungal sp. ARIZ and *T*. *atroviridae*, were less abundant, but were recovered with all three approaches as well (i.e., cultivation, environmental DNA, and RNA). It was not possible to recover *T*. *spirale* by cultivation, but was recovered from both the DNA and RNA-based approaches. Other fungal species, *A*. *hongkongensis, H*. *lixii, C*. *folicola, M*. *elongata,* and *Mortierella* sp. were present only in the DNA library and *T*. *velutinum* was recovered only in the RNA library.

### Terrestrial Fungi

In terrestrial soil, cultivation methods revealed five fungal taxa, comprising *A*. *furtiva, T*. *atroviridae, Penicillium canescens, H*. *lixii, and Cryptococcus podzolicus* (Table 2). Our DNA-based analysis of the same soil sample using a clone library (*n* = 100) yielded nine phylotypes (Supplementary Fig. 1). A BLAST search of the NCBI GenBank indicated that almost over 85 % of these showed affiliation with *A*. *furtiva*. The remaining phylotypes were affiliated with *T*. *velutinum* (4 %), *M*. *elongata* (3 %), *T*. *spirale* (3 %), *H*. *lixxii* (2 %), *Trichoderma koningiopsis* (1 %), *T*. *atroviridae* (1 %) and *Trichoderma tometosum* (1 %). The second library, constructed using cDNA synthesized from environmental RNA revealed 13 phylotypes, delineated as mentioned above. The BLAST search indicated that almost over 90 % of the sequences were affiliated with *A*. *furtiva*. The remaining phylotypes showed affinities with *H*. *lixxi* (3 %), *T*. *velutinum* (1 %), *Trichoderma ovalisporum* (1 %), *T*. *spirale* (1 %), *Trichoderma* sp. (1 %), *Aspergillus* sp. (1 %), *M*. *elongata* (1 %), and *Mortierella* sp. (1 %).

**Table2:** Summary of the relative abundance of fungal phylotypes in environmental RNA and DNA clone libraries of fungal ITS loci from the terrestrial soil of Tai Po Kau forest

S.No.	Best BLAST match	Culture	DNA (%)	RNA (%)	Sample label culture/DNA/RNA	Accession no.
Ascomycota						
1	*Anguillospora furtiva* (AY148107)	√	85	90	7PDA3/7DNA11/7cDNA36	JF831451
2	*Trichoderma koningiopsis* (AB568478)	×	1	0	-/7DNA13/-	JF831495
3	*Trichoderma atroviride* (HQ259980)	√	1	0	-/7DNA119/-	JF831471
4	*Trichoderma tometosum* (AY605737)	×	1	0	-/7DNA107/-	JF831474
5	*Trichoderma velutinum* (HM176574)	×	4	1	7DNA137/7cDNA7	JF831481
6	*Trichoderma ovalisporum* (EU280118)	×	0	1	–	–
7	*Trichoderma spirale* (EU280068)	×	3	1	-/7DNA31/-	JF831480
8	*Trichoderma* sp. (GQ497168)	×	0	1	-/7cDNA25	JF831478
9	*Aspergillus* sp. (HM573343)	×	0	1	–	–
10	*Penicillium canescens* (FJ439586)	√	0	0	7MEA3/-	JF831461
11	*Hypocrea lixii* (AB570245)	√	2	3	7MEA1/7DNA19/7cDNA75	JF831468
Basidiomycota						
12	*Cryptococcus podzolicus* [FN428924]	√	0	0	–	–
Fungi *incertae sedis*						
13	*Mortierella elongata* (AB542111)	×	3	1	-/7DNA75/7cDNA30	JF831499
14	*Mortierella* sp. (EU877758)	×	0	1	–	–

The polyphasic nature of our investigation on terrestrial soil indicated that the most common fungus was *A*. *furtiva*, which was dominant and recovered in all the three approaches. However, *H*. *lixii* was less abundant, but recovered with all three approaches as well. Three fungal species, *T*. *velutinum, T*. *spirale*, and *M*. *elongata* were not recovered through cultivation; however, they were recovered from DNA- and RNA-based clone libraries (Table 2). *Trichoderma koningiopsis* and *T*. *tometosum* were detected only in the DNA clone library and not in the RNA clone library. Similarly, the fungal species *T. ovalisporum, Trichoderma* sp., *Aspergillus* sp., and *Mortierella* sp. were detected in the RNA library alone and were absent in the DNA library.

**Fig. 2:**
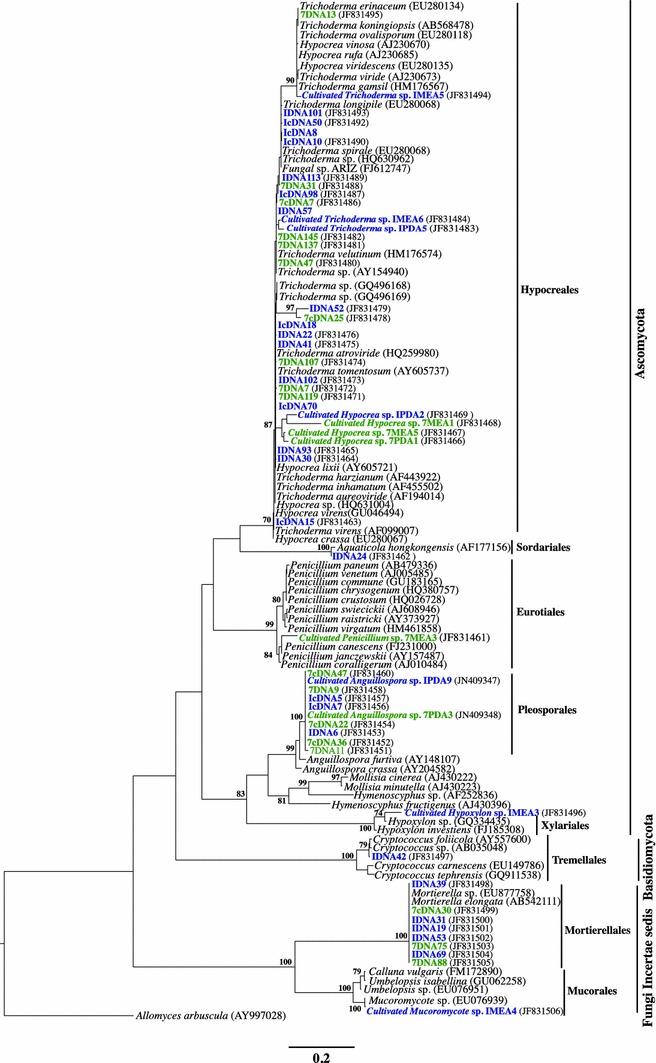
The maximum likelihood tree generated on sequence analysis on dataset obtained from ITS region (ITS1—5.8SrRNA gene—ITS2) of cultivated strain of Tai Po Kau forest soil, cDNA, and DNA phylotypes from clone libraries respectively, *blue color* (fresh water sediment), *green color* (terrestrial soil). *Bootstrap values*, given as percentage of 1,000 replicate trees, are indicated for branches supported by more than 70 % of the trees. The *scale bar* represents 0.2 nucleotide change per position (Color figure online)

Phylogenetic analyses of environmental phylotypes and cultivated strains from freshwater sediments and soil of Tai Po Kau forest resolved unambiguously into Ascomycota, Basidiomycota, and Fungi *incertae sedis* clades among orders: Pleosporales, Hypocreales, Sordariales, Eurotiales, Xylariales (Ascomycota), Tremellales (Basidiomycota), Mortierellales, and Mucorales (Fungi *incertae sedis*) (Fig. 2). Our combined direct cultivation and indirect DNA- and RNA (cDNA)-based approaches clearly detect differences between cultivable, total, and putatively active fungi. The dominant taxa recovered from the soil and sediment samples were *Anguillospora* taxa and species of this genus, which have previously been recovered by means of traditional baiting and laboratory incubation techniques from Tai Po Kau forest stream [9]. The remaining fungi were *Hypocrea/*–*Trichoderma, Hypoxylon, Aquaticola, Penicillium, Aspergillus*, Fungal sp. ARIZ, *Cryptococcus, Mortierella* and unidentified Mucoromycotina.

Overall, this study indicates that the fungal species represented in the forest soils belong to diverse functional ecological groups (parasitic, saprobic, and mutalistic). Furthermore, from the RNA-based analysis it is evident that in the fresh-water sediments, *Anguillospora*, *Trichoderma*, and Fungal sp. ARIZ were the active fungal species due to their relative abundance while in the terrestrial soil, the active species were *Anguillospora, Hypocrea*/*Trichoderma*, and *Mortierella*. Phylogenetic analysis for Ascomycota not only resolve taxonomy but also show some interesting anamorph/-teleomorph trends between *Hypocrea/*–*Trichoderma*. Previous studies [13] have revealed connections between *H*. *lixii* (teleomorph) and *T*. *harzianum* (anamorph), which is also represented in this study (Fig. 2). DNA- and RNA-based community profiling is not new to forest soil ecology. Recently, these approaches were applied to soil microbial ecology for the study of active bacterial and fungal communities [6]. The recovery of saprotrophic species from terrestrial soil; for instance, the basidiomycetous yeast, *Cryptococcus podzolicus* (well known for the decomposition of litter in forest), overall highlights the importance and ecological role of these fungi in the forest ecosystem.

## Conclusion

Our results showed that first, there is a marked variation in fungal-taxa recovery between traditional cultivation versus environmental DNA/–RNA signatures. Second, the variation in DNA and RNA fungal signatures indicate the potential difference between recoverable fungi and those that are active in the environment. The sequences obtained in this study help resolved taxonomy through traditional cultivation—environmental DNA- and RNA-based strategies. In the future, addressing further biases inherent in PCR-based recovery, and encouraging robust hierarchical sampling regimes, will improve our ability to estimate environmental diversity of fungi.

## Electronic Supplementary Material

Below is the link to the electronic supplementary material.

Supplementary material 1 (PDF 672 kb)
