# Supplementary material for: Comparison of DNA and RNA, and Cultivation Approaches for the Recovery of Terrestrial and Aquatic Fungi from Environmental Samples
Source: Curr Microbiol. 2012 Oct 27;66(2):185–91. doi: 10.1007/s00284-012-0256-7 (PMC3543764; doi:10.1007/s00284-012-0256-7)
Supplement: Supplementary file 1 — Supplementary material 1 (PDF 672 kb) [file 284_2012_256_MOESM1_ESM.pdf]

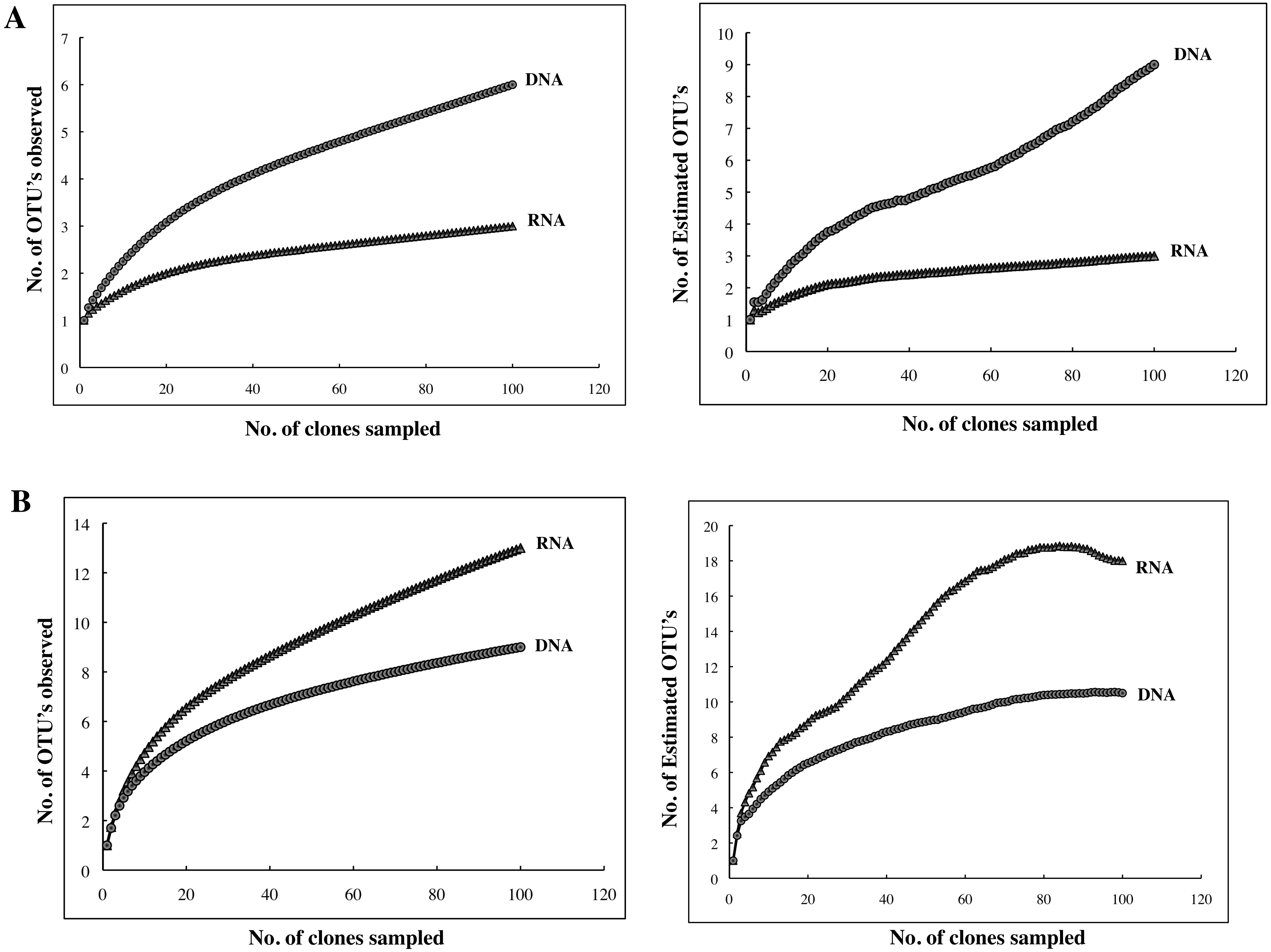

Supplementary Fig. 1. (A) Rarefaction and Chao1 estimates for DNA and RNA (cDNA) clone libraries for fresh water sediment samples. (B) Rarefaction and Chao1 estimates for DNA and RNA (cDNA) clone libraries for forest soil samples.
